# In silico modeling of oxygen‐enhanced MRI of specific ventilation

**DOI:** 10.14814/phy2.13659

**Published:** 2018-04-16

**Authors:** Wendy Kang, Merryn H. Tawhai, Alys R. Clark, Rui C. Sá, Eric T. Geier, G. Kim Prisk, Kelly S. Burrowes

**Affiliations:** ^1^ Auckland Bioengineering Institute University of Auckland Auckland New Zealand; ^2^ Department of Chemical & Materials Engineering University of Auckland Auckland New Zealand; ^3^ Department of Medicine University of California San Diego La Jolla California

**Keywords:** Computational model, oxygen‐enhanced, proton MRI, specific ventilation

## Abstract

Specific ventilation imaging (SVI) proposes that using oxygen‐enhanced 1H MRI to capture signal change as subjects alternatively breathe room air and 100% O_2_ provides an estimate of specific ventilation distribution in the lung. How well this technique measures SV and the effect of currently adopted approaches of the technique on resulting SV measurement is open for further exploration. We investigated (1) How well does imaging a single sagittal lung slice represent whole lung SV? (2) What is the influence of pulmonary venous blood on the measured MRI signal and resultant SVI measure? and (3) How does inclusion of misaligned images affect SVI measurement? In this study, we utilized two patient‐based in silico models of ventilation, perfusion, and gas exchange to address these questions for normal healthy lungs. Simulation results from the two healthy young subjects show that imaging a single slice is generally representative of whole lung SV distribution, with a calculated SV gradient within 90% of that calculated for whole lung distributions. Contribution of O_2_ from the venous circulation results in overestimation of SV at a regional level where major pulmonary veins cross the imaging plane, resulting in a 10% increase in SV gradient for the imaging slice. A worst‐case scenario simulation of image misalignment increased the SV gradient by 11.4% for the imaged slice.

## Introduction

The efficiency of gas exchange in the lung is governed by the regional matching of alveolar ventilation (*V*_A_) and perfusion (*Q*). Mismatch between *V*_A_ and *Q* is the most significant contributor to impaired gas exchange in disease (Rodriguez‐Roisin et al. 2009). At present, imaging of pulmonary blood flow using MRI is well established (Hopkins et al. 2007; Henderson et al. 2009), but imaging of ventilation is less well developed. Specific ventilation (SV) is a measure of the efficiency of lung ventilation, defined as the ratio of the volume of fresh gas entering a region to its end‐expiratory volume (Sa et al. 2010). In this study, we assess a functional imaging technique that involves the use of oxygen‐enhanced proton MRI to quantitatively measure SV in the lungs (Sa et al. 2010, 2014). While MR‐based measurements of ventilation distribution using hyperpolarized gases are now becoming common, for the most part these do not produce quantitative results, the exception being work that has used short multiple‐breath washouts of hyperpolarized gases (Horn et al. 2014; Hamedani et al. 2016, 2017).

The ^1^H‐MR‐based specific ventilation imaging (SVI) technique exploits the paramagnetic properties of oxygen (O_2_), where local change in O_2_ concentration ([O_2_]) alters the spin‐lattice relaxation time constant (T1) and thus alters the signal intensity of appropriately timed inversion recovery MR images (Ohno and Hatabu 2007). In a typical implementation, subjects alternate between breathing room air and 100% O_2_ for blocks of 20 breaths, repeated five times with one 40‐breath O_2_ block at the end (a total of 220 breaths), and the MR measurements track signal changes during the washin and washout of the O_2_. The rate of change of local MR signal over time is used to estimate SV within each image voxel.

Most SVI studies to date have imaged only a single (15 mm thick mid‐sagittal) slice from the right lung representing approximately 8% of the lung volume at functional residual capacity (FRC) (Sa et al. 1985, 2010, 2014) leading to the obvious question: Are the measurements of SV in a single sagittal slice representative of SV (gravitational distribution and heterogeneity) in the whole lung? Secondly, an important assumption of the SVI technique is that local lung tissues are in equilibrium with the surrounding P_A_O_2_. However, in reality some signal will be produced from the O_2_ contained within the pulmonary arteries and veins, flowing from distal parts of the lung, and this blood will not necessarily have the same po_2_ as the local tissue being interrogated. Finally, the methodology of SVI acquisition as currently implemented requires a voxel‐by‐voxel correlation from 220 MR images (one image per breath), acquired over a period of about 18 min. Although subjects are trained to gate their breathing with the MRI acquisition procedure (aiming for a natural breathing rate and tidal volume) and to briefly suspend breathing at FRC during image acquisition, some misalignment of lung tissue across breaths is inevitable. Image registration has not been systematically applied to SVI studies to date (Sa et al. 2010, 2014), and although images that are significantly displaced are discarded, an examination of how in‐plane image misalignment affects the resulting SVI measurement is required.

In this study, we conducted a theoretical assessment of how well SVI measurement as typically performed represents SV distributions in the normal human lung, using an anatomically realistic computational model framework. This model contains the full conducting airway tree and the accompanying pulmonary arteries and veins. Fluid flows (ventilation and blood flow) were predicted within the 1D network models. The specific questions we set out to answer were:

1. How representative is the SV distribution determined from a single 15 mm slice of the SV distribution in the whole lung?
2. What is the contribution of pulmonary venous blood to the SV signal? How does this contribution vary as a function of slice location?
3. What is the potential impact of in‐plane misalignment of images across breaths on the SVI measurement?

## Methods

### The in silico model

**Figure 1:**
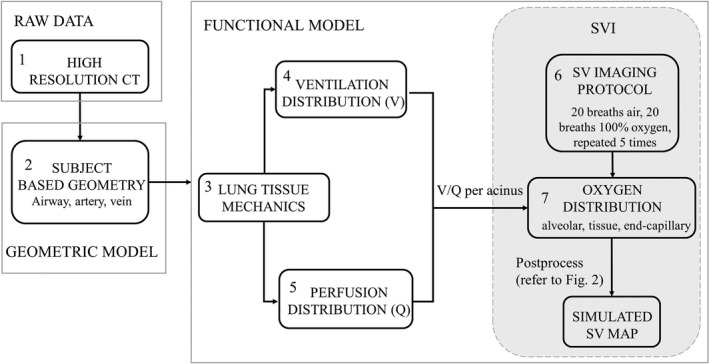
Schematic of model components applied to simulate the specific ventilation (SV) imaging protocol. Two subject‐based geometric models (1 male, 1 female) were derived from CT scans (steps 1, 2). Functional models based on the underlying biophysical laws were used to predict tissue deformation (step 3), regional ventilation (step 4), perfusion (step 5) and resultant gas exchange per voxel (step 7). The SV imaging protocol (step 6) was simulated and the resultant O_2_ distribution within lung tissue and blood further processed to generate a voxelized SV image.

In this study, we applied a similar lung modeling framework to that of Kang et al. (2017) consisting of a model of tissue mechanics, ventilation, blood flow, and gas exchange to simulate the distribution and exchange of oxygen during tidal breathing in supine subjects The various modeling steps are illustrated in the schematic in Figure 1. Each of these modeling components has been published previously. A short summary of the model components is provided below, with the key aspects and equations used reiterated in the Appendix A.

Unless otherwise indicated, no significant changes have been made to the methodology of the models. In brief, we used two subject‐based geometric models of the conducting airways and accompanying pulmonary blood vessels. High‐resolution CT scans of two healthy subjects (S1: female, 22 years old, 57.1 kg, 1.60 m tall, 2.64 L at FRC and S2: male, 21 years old, 80.7 kg, 1.78 m tall, 3.62 L at FRC) from the Human Lung Atlas database (University of Iowa (Hoffman et al. 2004)) were used to create the models. Central airways (to approximately 6 generations) and lobar surfaces were segmented from the CT scan at FRC using the custom‐built software “PASS” (Pulmonary Analysis Software Suite, University of Iowa). The airway segmentations were automatically analyzed using PASS to calculate the centerlines of the airways, location of bifurcations, and airway radii. Geometry of the centerlines of central arterial and venous vessels (to approximately 4 generations) were manually estimated from the volumetric CT. Additional branches, down to the level of the gas exchange tissue, were created using a volume‐filling branching method (Tawhai et al. 2004). The networks are spatially distributed within the 3D lung volume but are described as 1D branches (lines) with associated diameter information. Distal to the CT‐derived branches, the vascular models had identical spatial distribution (length and *x*,* y*,* z* coordinates) as the airway tree (although diameters differed), meaning that all three branching trees terminated at the same physical location (feeding into a single acinus). Each lung consisted of approximately 32,000 branches (for each of the three networks – airways (Tawhai et al. 2004), arteries and veins (Burrowes et al. 2005)) and 16,000 acini. The use of this conducting airway geometry in which air flow (ventilation) and O_2_ concentration are solved accounts for the effect of different regional dead space on the specific ventilation of individual gas exchanging units.

A model of lung tissue mechanics (Tawhai et al. 2009) was solved to predict the gravitational distribution of lung tissue volumes and the resulting acinar volumes at FRC (see Appendix: lung tissue mechanics). Airways and pulmonary vessels were embedded in the tissue model and deformed with it. Simulations were all conducted in the supine posture, consistent with the imaging methodology. A model of ventilation was applied within the conducting airway network to obtain a time‐averaged distribution of ventilation (*V*_A_) within each acinar unit in the lung model (Swan et al. 2012) (see Appendix: ventilation). Output of the ventilation model gives breath‐averaged flow to each acinar unit and from this the simulated **true** value of SV was calculated (in terms of SV = volume of gas entering region (∆*V*_A_)/end‐expiratory volume of gas in the same region (*V*_0_)). The simulated SVI (described by the process in Fig. 1) measurements were compared against this simulated true SV. A steady‐state blood flow model throughout the full pulmonary circulation – arteries, capillaries, veins – was solved to predict the regional distribution of blood flow (*Q*) and blood volume to each acinus (Clark et al. 2011) (see Appendix: perfusion). The resultant distribution of *V*_A_/*Q* at each acinus was utilized in a model of gas exchange to predict the regional partial pressure of oxygen (PO_2_, where P_c_O_2_, P_A_O_2_, P_a_O_2_, P_t_O_2_, and P_v_O_2_ refer to end‐capillary, alveolar, arterial, tissue and venous partial pressures, respectively) (Swan and Tawhai 2011). Details of the calculation of this can be found in the Appendix: [O_2_] distribution in airways and vasculature. In addition, study‐specific modeling parameters are listed in Table A1.

### Simulating the specific ventilation imaging (SVI) protocol

The SVI protocol, described in (Sa et al. 2010, 2014), was applied to the model. The virtual lung cycled through 20 tidal volume breaths of room air and 20 breaths of 100% O_2_ repeated five times. An additional 20 breaths of 100% O_2_ were added at the end of the final cycle of O_2_ as in the imaging protocol (total 220 breaths). All simulations were performed at a constant tidal volume of 650 mL. The model predicted the distribution of O_2_ throughout the system – air, tissue, and blood. Since pulmonary arterial blood is spatially uniform in terms of PO_2_ (all mixed venous blood is the same), it would resemble an offset in the amplitude of the signal. Therefore, for simplicity, we excluded this effect. The following four steps were followed to create the SV image from the model O_2_ distribution:

1. The contribution of O_2_ from tissue (alveolar and end‐capillary blood O_2_)
2. The contribution of O_2_ from pulmonary venous blood
3. Voxelization (the process of splitting the model up into voxels)
4. Translation of the [O_2_] time series to the SVI measurement.

Each of these steps is described in more detail in the following sections. This process enables us to quantify the separate contributions of [O_2_] from the tissue (what we ideally want to measure) and from the venous blood (considered to be artifact or unwanted signal) to the resultant SV image.

#### The contribution of O_2_ from the tissue

**Figure 2:**
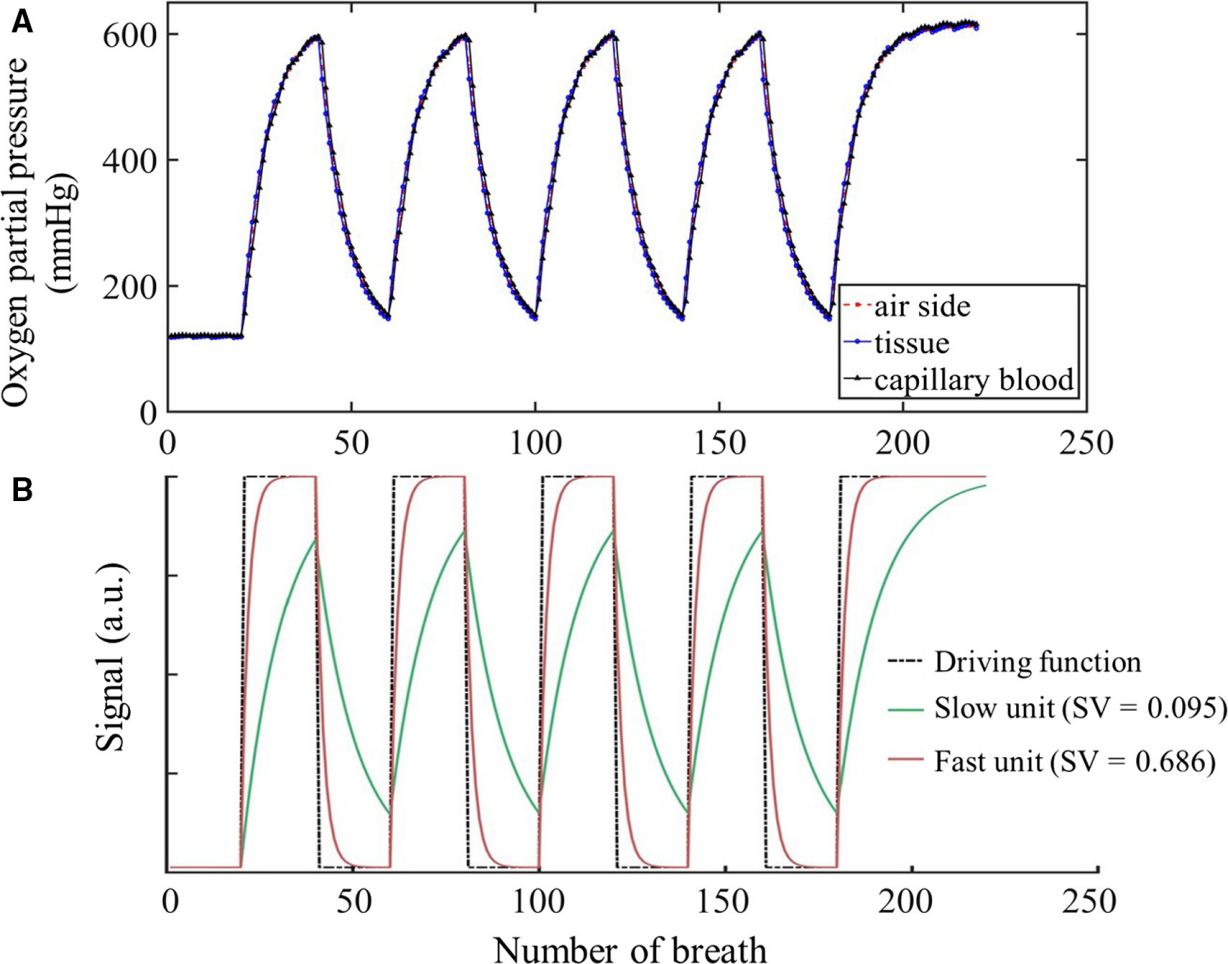
(A) Example of a breath‐by‐breath trace of model outputs for a single gas exchange unit (SV = 0.193), consisting of air side, tissue and capillary blood compartments. Oxygen partial pressure is reported on the *y* axis, and is translated and combined into [O_2_] for each voxel during postprocessing. The time evolution of partial pressures for the three compartments are superimposed onto each other and not readily separable. (B) Characteristic response curve of selected specific ventilation (SV) units during the SV imaging protocol. Note, response signal on the *y* axis of the characteristic response curve represents the local maximum, directly related to the equilibrated [O_2_] for that unit. Units with higher SV equilibrate faster with a steeper gradient (red line), while units with lower SV equilibrate slower (green line). Maximum correlation between acquired unit signal (A) and characteristic response (B) for an SV unit gives the MRI‐inferred SV (SVI measurement) for that unit.

In the model, each terminal node of the airway tree corresponds to a single acinar unit. The oxygen concentration [O_2_] within the capillary blood and surrounding tissue of each acinar unit was recorded at the end of each breath to provide a time series of [O_2_] values which is reflected in the time series of MR signal (in arbitrary units, a.u.), see Figure 2A.

#### The contribution of O_2_ from venous blood

Dissolved molecular O_2_ shortens the T1 relaxation time and, therefore, dissolved O_2_ in the oxygen‐rich pulmonary veins is expected to influence the signal intensity of T1‐weighted MR images. The venous blood contribution does not necessarily correlate with the local SV but instead adds confounding signal to the MR image from distant parts of the lung. In order to determine the complete SVI measurement, the distribution of [O_2_] throughout the venous network at the end of each breath was included in our analysis. The venous contribution was calculated by taking the end‐capillary O_2_ recorded at the end of each breath (calculated using the gas exchange model, see Appendix A for more details) and summing this O_2_ at confluent bifurcations along each venous pathway from capillary to the heart. The time lag in O_2_ traveling along each element of the venous pathway was introduced using the blood flow rate and the distance that blood needed to travel to reach the venous element. Thus, [O_2_] at each venous bifurcation is the flow weighted sum of time‐lagged O_2_ from its respective upstream capillaries.

#### Voxelization

To allow comparison with MR image‐based voxel measurements in previous publications (Sa et al. 2010, 2014) the model was partitioned into equally sized voxels (1.6 × 1.6 × 15 mm) in the sagittal plane. For the tissue analysis, the acinar volumes were split into voxels. The [O_2_] within each voxel was summed in proportion to the acinar unit volumes predicted at FRC (tissue mechanics model) that occupied the voxel.

For the venous blood, the venous network was also partitioned into voxels. For this calculation, each venous branch was represented as a 3D (volumetric) cylinder applying the respective branch radius wrapped around the 1D centerline of the branch. In order to attribute venous signal across multiple voxels (particularly important for large vessels that span more than one voxel), each venous branch (cylinder) was represented as a dense cloud of uniformly spaced data points that split the element into small volumes of 1 × 10^−3^ mm^3^ each. The number of data points distributed to each voxel was considered proportional to the volume each venous element occupies in the voxel, and thus corresponded to the amount of venous signal each voxel received. The voxelized venous signal was added to the tissue signal. Voxels less than 50% filled with lung tissue were excluded from further analysis, in essence removing partial volume effects at the lung edge. After these steps, the resulting sequence of virtual “images” were comparable to typical MR image resolution. This combined MR signal sequence was then translated to SVI measurement, described in the following step.

#### Translation of the [O_2_] time series to the SVI measurement

SV is defined as the volume of “fresh” gas going into a unit normalized by its end‐expiratory volume, and so describes the rate of equilibration of a unit upon change in inspired [O_2_] (FIO_2_). For two identically sized units, the unit with more fresh air/oxygen delivery (higher SV) equilibrates at a faster rate than the unit with less delivered fresh air/oxygen. The varying rates of equilibration for different SV units form the characteristic response curves described by Sa et al. (2010) and illustrated in Figure 2B. In order to translate the simulated [O_2_] time series response into SV measurement for each voxel, a similar approach to Sa et al. (2014) was applied. A library of characteristic response curves was generated for 50 SV units, spanning a SV range of 0.01–10 with 15% increment in SV in each unit. The time sequence for each voxel within the lung model was correlated with each of the characteristic response curves in the library. The highest significant correlation between the time‐course of a voxel and one of the 50 SV unit response curves provided the SV estimate for that voxel, and was kept if the Spearman correlation was significant (*P* ≤ 0.05). If the highest correlation was not significant (*P* > 0.05), the voxel was discarded from further analysis. Approximately 2% of the voxels were discarded as a result of this step.

From its definition, SV throughout the lung can be directly calculated from alveolar tidal volume divided by EELV (end‐expiratory lung volume); these variables are outputs of the ventilation and tissue mechanics models, respectively. This calculated SV is considered as the *simulated true SV*, against which the simulated SVI measurements are compared. In the first step of our analysis we compared the *simulated true SV* (gradient and coefficient of variation) with the SVI measurement obtained from simulated MR signal.

### Single slice versus whole lung comparisons

To avoid cardiac motion artifacts and maximize the sample in the anterior‐posterior dimension, while avoiding major hilar vessels, the typical SV imaging slice has been selected in the mid‐sagittal line of the supine right lung. The in silico lung was divided into 15 mm thick sagittal slices, and a similarly placed slice in the mid‐sagittal line was selected as the MR equivalent region of interest (ROI) to compare against other slice locations. The SVI quantification in this single slice was compared against that of the whole lung (both left and right lungs).

### Estimating the impact of image misalignment

A single mid‐sagittal slice of the right lung (the same as used in *Single slice vs*. *whole lung comparison*) was taken where the [O_2_] solution within each acinus of the full lung model was recorded across the 220 breaths. To test the potential impact of in‐plane misalignment, we used the EELV percent volume change (the effective misalignment) recorded from a real MR SVI experiment in which there was poor reproducibility of alignment. Each “image” in the modeling sequence (including venous signal) were purposely misaligned by fixing the apex side of the lung slice and shifting the side representing the diaphragm edge downward along the cranio‐caudal axis, until relative volume change for each image aligned to within 10% of that specified by the measured deformation. The second subject (S2) used the same measured EELV deformation time series, randomly shuffled. This simulation mimics the situation where a subject was unable to maintain a constant EELV during the SVI protocol. Both the location and size of the acini were updated with the change in simulated EELV slice volume, and resultant shift of signals were subjected to voxel analysis using the initial voxel grid (from step *2. Voxelization*). Portions of the lung that appear outside of the initial lung boundary due to the in‐plane misalignment simulation were discarded. The resultant voxelized signals were then converted to the SVI measurement.

### Analysis of SV and SVI distribution and heterogeneity

To allow quantitative comparison against typically used measures, distributions of SV are presented in terms of a gravitationally dependent gradient and coefficient of variation (COV=standard deviation/mean). The supine lung was divided into 10 mm thick iso‐gravitational sections, and the mean value and standard deviation (SD) per gravitational section were calculated. Gradient measures were then obtained by linear regression of mean SV within a section against the vertical height of the lung.

## Results

### Simulated SVI measures versus model simulated true SV

The gradients, mean values, and coefficients of variation in simulated true SV and SVI measurement (including both tissue and venous blood O_2_) for a single mid‐sagittal slice are provided in Table 1. For both subjects, simulated SVI‐based distributions compared well to the simulated true SV in terms of both gradient and COV values, and suggests the model simulations replicate the SVI protocol and reproduce the simulated true SV.

**Table 1:** Comparison of simulated true SV and model SVI measurement in a single mid‐sagittal slice. The SVI measurement that measured when including contributions from tissue and venous blood, and is thus equivalent to the signal that would be obtained in the actual measurement

	S1 (female)		S2 (male)	
	Simulated true SV	SVI measurement	Simulated true SV	SVI measurement
Gradient (/cm)	−0.017	−0.013	−0.016	−0.018
Mean value	0.43	0.47	0.31	0.35
COV (%)	17	16	16	16

### Is a single mid sagittal slice representative of whole lung SV?

**Figure 3:**
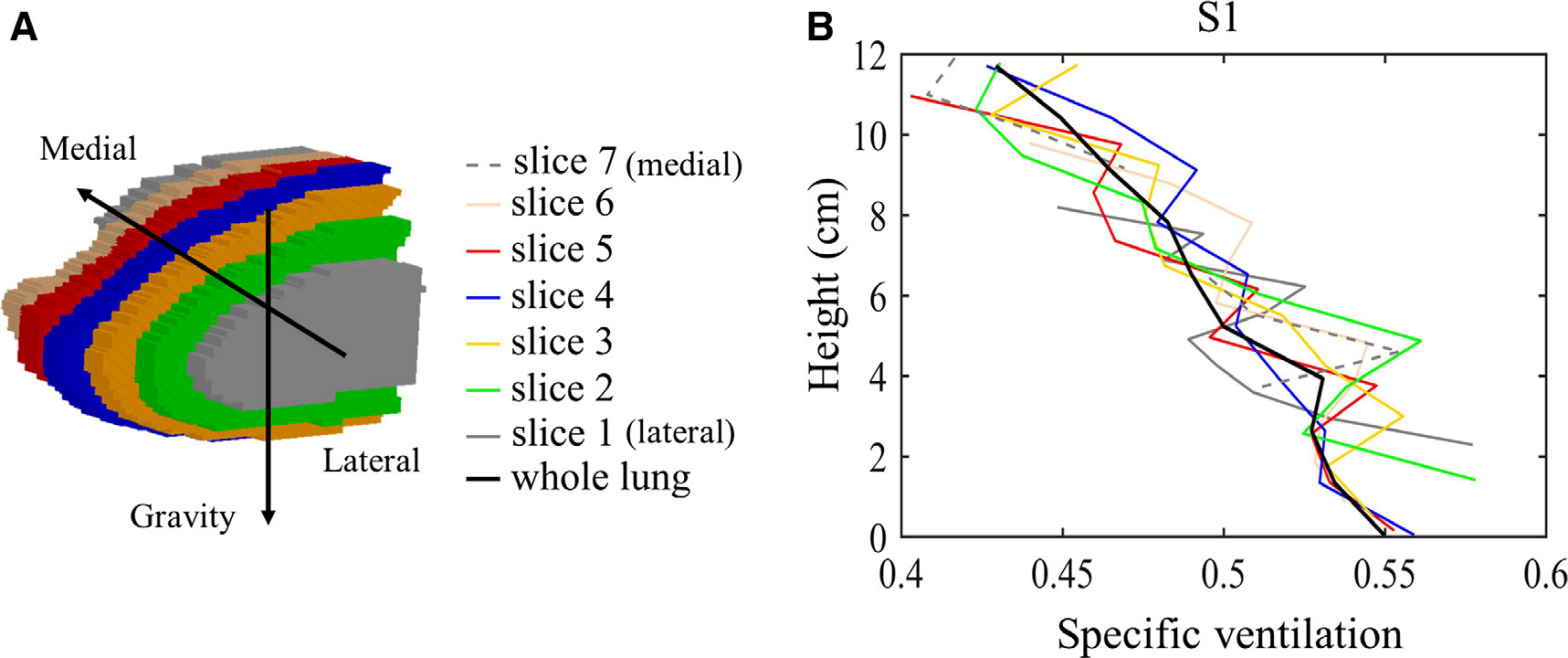
Gravitationally dependent specific ventilation distribution in multiple slices through the right lung. SVI measurements were derived from simulated MR signal from both tissue and veins. (A) Shows sagittal slice locations in the right lung, and (B) shows corresponding SV vertical gradients per slice, plotted as mean SV in isogravitational sections versus absolute lung height for both subjects. It should be noted that “whole lung” refers to both left and right lungs.

The SVI protocol was simulated for the entire lung, on a per slice basis, and results for left and right lungs were not significantly different for the two healthy subjects simulated. For the sake of simplicity and consistency with previously reported SVI measures, we utilize only the right lung in the following analysis. Each right lung was divided into 7 slices, and Figure 3 shows the gravitationally dependent measured SV distributions (following steps 1–4 from the methods section and including contributions from both tissue and venous blood) of all slices for subject S1 (Fig. 3B) with corresponding sagittal plane locations (Fig. 3A). Moving from the lung periphery to more central locations increased the extent of lung height and volume of lung each slice accounted for. The most peripheral slices (slices 1 and 7, colored gray in Fig. 3) only included 2.1–3.9% of the total lung volume each, and had gravitational gradients that had a limited extent compared to the rest of the lung slices due to the shape of the lung. For both subjects, the more central slices (Burrowes et al. 2005; Fain et al. 2008; Clark et al. 2011; Hamedani et al. 2016, 2017) showed similar gravitational distributions, and compared well with the whole lung gravitational distributions (black, Fig. 3B). Note that the SV distribution for the whole lung includes both the left and right lungs but the per slice trends illustrated are for the right lung only.

Slice 4 (blue, Fig. 3) is the mid‐sagittal slice, comparable to that used in a typical SVI experiment, accounting for 11.5 and 9.5% of subject S1 and S2's total right lung volume, respectively. The linear gradients for the data from the mid‐sagittal slice in Figure 3 (blue) are shown in Table 2 for each subject. Table 2 compares the overall measurement parameters (gradient, mean value, and COV) for SVI (including tissue and venous contributions) in a single mid‐sagittal slice and the whole lung for both subjects, S1 and S2. These values are shown alongside the simulated true SV from the whole lung model. The SVI measurement slightly underestimated the gravitational gradient for subject S1 (compared to whole lung, simulated true SV), but the gravitational gradient from the SVI simulation of whole lung and mid‐sagittal slice were similar. For subject S2 (the larger, male subject) the gradients were similar in all cases.

**Table 2:** Comparison of SVI measurement (gravitationally dependent gradient, mean and coefficient of variation, COV, of SVI) in a single mid sagittal slice with that of the whole right lung (values rounded to 2 or 3 significant figures). SVI values include signal contribution from both tissue and venous blood

	S1 (female)			S2 (male)		
	Simulated true SV	Model SVI measurement		Simulated true SV	SVI measurement	
	Whole lung	Whole lung	Mid‐sagittal slice	Whole lung	Whole lung	Mid‐sagittal slice
Gradient (/cm)	−0.017	−0.012	−0.013	−0.017	−0.016	−0.018
Mean value	0.45	0.48	0.47	0.35	0.37	0.31
COV (%)	18	25	16	16	21	16

**Figure 4:**
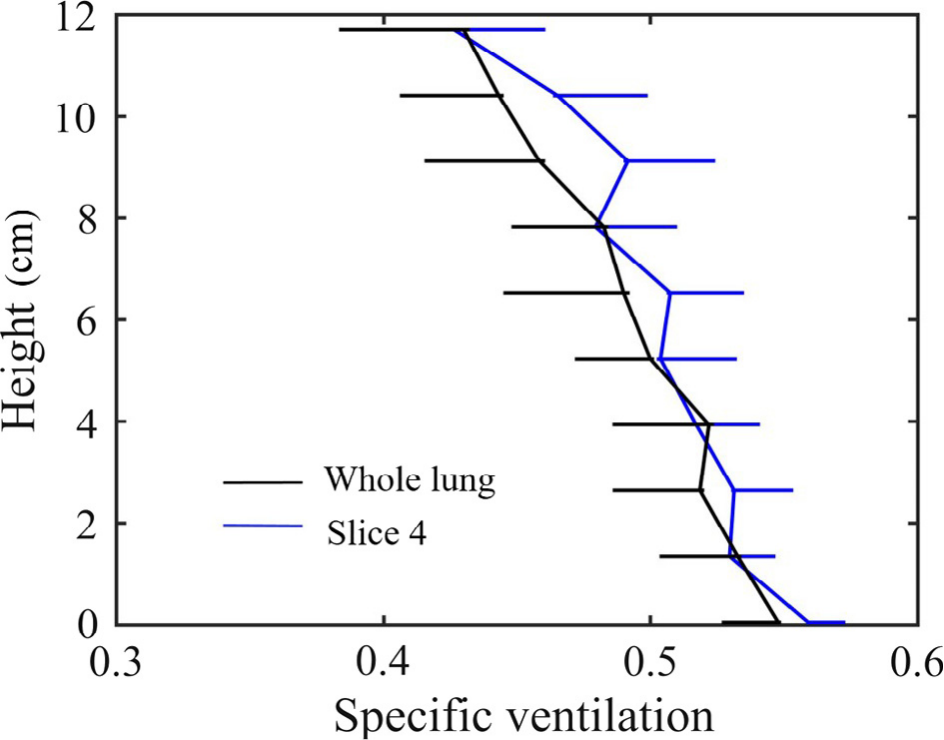
Comparison of the gravitationally dependent specific ventilation gradient in the mid sagittal slice from the right lung compared to that measured in the entire lung (for subject S1). Upper bound error bars (+Standard deviation, SD) are shown for measurements from the mid sagittal slice, and lower bound error bars (−SD) are shown for measurements based on the entire lung.

A closer comparison between the gradient from the mid‐sagittal slice against whole lung is shown in Figure 4 for subject S1. While gradients were not significantly different between the mid‐sagittal slice and the whole lung, greater heterogeneity in the iso‐gravitational direction was evident in the whole lung SVI measurement, a result also shown in Table 2.

### Contribution of pulmonary venous blood

**Figure 5:**
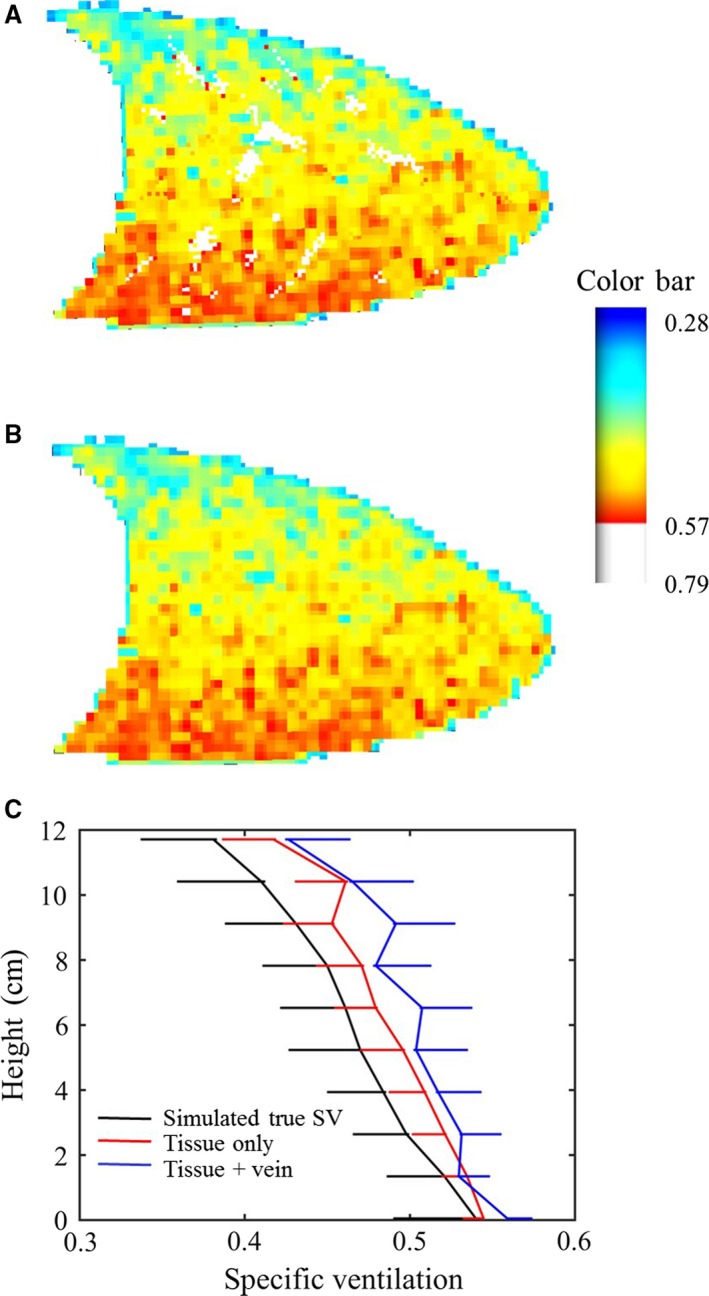
Simulated specific ventilation (SV) in 15 mm thick mid‐sagittal slices through the right lung for subject S1 (female). (A) SV map derived from tissue and venous signal contributions and (B) SVI measurement after removing the venous signal. (C) Height plots of the SVI distribution comparison for SV maps in (A) and (B). Upper bound error bars shown for SVI measurements from tissue and venous signal, while lower bound error bars are shown for the simulated true SV and tissue signal only cases. Note: (A) and (B) used the same color scale, indicated by the color bar at the top right of the figure.

Figure 5 displays the simulated SV map, for subject S1, for the mid‐sagittal slice with (Fig. 5A) and without (Fig. 5B) the pulmonary venous signal contribution, to show its impact on SV distribution and heterogeneity. In both cases there is a clear gradient with higher SV in the gravitationally dependent region and considerable heterogeneity throughout the lung. Comparing Figures 5A and B, localized regions of high SV values (indicated in white, with SV values >0.57, see the color bar in the plot) are evident in Figure 5A. These high SV values emanate from large venous vessels and account for approximately 4.2% of voxels within the slice.

Figure 5C shows the measured SV distribution for the imaged mid‐sagittal slice, calculated from a combination of tissue and venous signal compared to measured SV calculated using the tissue signal only. Measured SV calculated using both tissue and venous signal corresponds to that obtained from an actual MR SV image. There was a slight increase in SV calculated from total signal (tissue and venous) in comparison with that calculated from using only tissue signal, which also served to increase the COV. This result is reiterated in Table 3, which presents the gradient values, mean SV and COV of SV for both subjects, illustrating the contribution of the venous blood to the measured SV in both subjects S1 and S2. There was a maximum increase in 13% in the gradient and 23% increase in coefficient of variation when comparing SV calculated from total signal (tissue and vein) with only tissue signal for the two subjects.

**Table 3:** Comparison of SVI properties (gravitationally dependent gradient, mean and coefficient of variation, COV, of SVI) including and excluding the contribution of venous signal (values rounded to 2 or 3 significant figures). Values presented are all for a single mid sagittal slice (slice 4 in the model)

	S1 (female)			S2 (male)		
	Simulated true SV	SVI measurement		Simulated true SV	SVI measurement	
		Tissue + vein signal	Tissue signal only		Tissue + vein signal	Tissue signal only
Gradient (/cm)	−0.017	−0.012	−0.013	−0.016	−0.018	−0.016
Mean value	0.43	0.49	0.47	0.31	0.35	0.34
COV (%)	17	16	13	16	16	13

We also incorporated the potential contribution of O_2_ from the deoxygenated arterial blood by adapting a similar approach to that of the venous blood. Oxygen partial pressure within the arterial blood was treated as a constant at 40 mmHg. As expected, arterial blood flow made little change to the signal, and did not alter the resultant SV distribution upon its inclusion to the simulated MR signal. Measured SV distribution including arterial signal is therefore not further reported here.

### The impact of image misalignment

**Figure 6:**
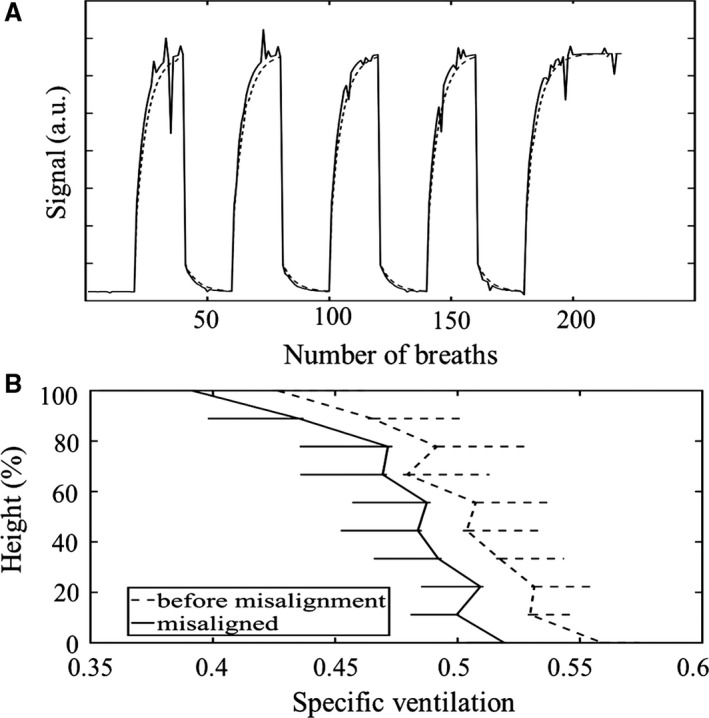
Comparison of effect of image misalignment on MR signal and resultant SV distribution. Results are shown for Subject S2, after artificially imposing misalignment of “images” using a set of EELV change calculations recorded in a real SV imaging experiment. (A) Breath by breath tracking of signal in a selected voxel before image misalignment (SV = 0.29) and after misalignment (SV = 0.23). (B) Gravitational distribution of SV in the mid sagittal slice before and after misalignment. Upper bound errors are shown for the case before misalignment simulation, and lower bound error bars shown for SVI from misaligned signal.

Overall, the misalignment procedure introduced a 9 ± 12% change in EELV onto each “image” of the total signal sequence for the mid‐sagittal slice of both subjects, with a maximum misalignment of 40%. A breath by breath tracing of the “before misalignment” and misaligned signal for a selected voxel is shown in Figure 6A. The effect of this misaligned signal on resultant SV distribution for the mid‐sagittal slice is shown in Figure 6B, as a function of SV plotted against percent of gravitational lung height. For both subjects, image misalignment resulted in a slightly reduced SV (from measured SV using both tissue and vein signal) across all gravitational sections. The SV underestimation was slightly greater in the posterior (gravitationally dependent) edge of the lung, resulting in a higher gravitational gradient. The effect of misalignment on measured SV was more prominent in subject S1 than subject S2, showing an increase in gravitational gradient and coefficient of variation of specific ventilation of 11.4 and 8.1%, compared to 4.9 and 5.1% for S2, respectively.

## Discussion

The use of O_2_ as a contrast agent in oxygen‐enhanced MRI has allowed for the application of clinically available proton MRI to functionally image the lung, which is a difficult to organ to image because of its low proton density environment in comparison to other body organs (Wild et al. 2012). The technique's applicability is potentially limited by confounding factors in the form of venous signal and image misregistration, and by potential sampling bias from imaging a small portion of the lung volume. In this study, we have examined the effects these factors may have on the quantification of SV in two patient‐based in silico lung models. To the best of our knowledge, this is the first study that is able to separately quantify the contribution of tissue O_2_ from venous O_2_ to MR‐measured SV, which is a physiologically relevant issue for all oxygen‐enhanced proton MRI‐based ventilation imaging methods, and of particular importance to the quantification of SV using SVI. Results from our analysis show that for the case of young healthy lungs, the current single centrally placed image slice is representative of whole lung SV distributions. Pulmonary venous O_2_ was found to create localized voxels with erroneously high SV (Fig. 5A for S1). Across the two subjects studied, pulmonary venous blood resulted in a maximal effect of a 6.5% increase in mean SV, it slightly increased the gravitational gradient (12.5%) and increased the heterogeneity (as measured here by the coefficient of variation, increase of 23%). Simulation of an exaggerated case of image misalignment shows measured SVI gradient is robust against a modest extent of image misalignment during image acquisition and results in a reduction in measured SVI.

### Simulated specific ventilation imaging versus simulated true SV

Overall, simulated SVI measurement (tissue signal only case) showed similar increase in specific ventilation toward dorsal lung as seen in the simulated true SV, but with a slightly decreased COV for both subjects simulated (Table 1). The decreased COV was expected for two reasons. The grouping of signals that actually exist on a continuous SV spectrum into discrete bins serves to reduce the COV. Secondly small errors associated with signal translation during the postprocessing phase (see *Translation of SVI measurement*, above) cause a unit to correlate slightly better with a neighboring unit than the simulated true SV unit. The error in estimation of simulated true SV distribution seen here is well within the magnitude of the reproducibility bias (2 ± 16%) in heterogeneity between two SVI experiments on the same subject quantified by Sa et al. (2014).

### Single slice versus whole lung SVI

As seen in Figure 3B, all medial slices gave similar estimates of the whole lung SV gradient. The gradient for the mid‐sagittal slice was slightly larger than for the whole lung (Fig. 4), likely because this mid‐sagittal slice is the tallest with the greatest amount of gravitational variation. The coefficient of variation estimated for the mid‐sagittal slice was slightly smaller than that calculated for the whole lung. While SVI maximizes sampling in the gravitational direction (sagittal plane) when using a mid‐lung slice as the ROI, only a small portion (15 mm in L‐R direction) of the lung is imaged and thus is expected to underestimate the SV heterogeneity compared to the whole lung estimate (Table 2). The result is consistent with a previous report that measured a 11 ± 19% higher standard variation for the whole lung Multiple Breath Washout technique compared to that obtained to single‐slice SVI across ten healthy subjects (Sa et al. 2014). This result suggests that information obtained from the current single slice imaging approach is generally representative of whole lung distributions when considering ventilation inhomogeneity in a healthy lung (Sa et al. 1985).

### Contribution from pulmonary venous blood

The SV distribution obtained for the mid‐sagittal slice including the pulmonary venous signal (Fig. 5) resembles the general patterns seen in a typical SV map from an SVI experiment (see, for example, Fig. 1B in (Sa et al. 2014)). Locations where large venous vessels crossed the imaging slice coincided with the highest SV units (clusters of white voxels, Fig. 5A). As a result, there is a larger variation in SV compared to that calculated using only tissue signal which increases the gravitational gradient in SV by 10 and 9%, and increases in COV by 23 and 19% for subjects S1 and S2, respectively (Fig. 5C and Table 3).

Overall, addition of signal from the pulmonary venous blood within each imaging slice resulted in only small over estimation of SV (Fig. 5). This is because SV corresponds to the rate of equilibration of the signal after a step change in F_I_O_2_ rather than the absolute signal intensity. Signal originating from the finer veins in the venous network may serve to increase the rate of rise in the MRI signal slightly providing a likely explanation for the small increase in SVI resulting from venous signal on overall the SVI measurement (Fig. 5C). Since pulmonary venous blood in the small veins likely results from gas exchange units in close proximity to the voxel of interest one would expect that the rate of increase in O_2_ in this blood would closely match that in the tissue in close proximity. However, for the larger pulmonary veins, the venous blood is likely dominated by high SV units which would also be expected to be high perfusion units, and thus result in an overestimation of SV compared to measured that from tissue signal only (Fig. 5).

### The impact of image misalignment

Simulated in‐plane image misalignment during the SVI protocol resulted in consistent underestimation in SV (Fig. 6B), an increase in the gravitational gradient, and an increased COV of the measured SV distribution. The response curve of a unit with higher SV is a rapid change from 21% O_2_ to equilibrium at 100% O_2_ values, while the response curve of a unit with lower SV is a slower progression toward equilibrium (see Fig. 2B). Thus, the overall signal turnover for that voxel is weighted toward the longer tail of the slower signal rise from the lower SV unit. This is illustrated in Figure 6A, where the voxel signal appears to rise more slowly toward equilibrium compared to the corresponding voxel signal obtained before image misalignment. The effect is to bring units of differing SV into the same imaging voxel, creating a pseudo‐unit, the signal from which is a mixture of the SV from the contributing units. The result is a bias toward the lower of the contributing SV values, which can be seen to occur across the entire vertical extent of the sagittal slice (Fig. 6B).

Our simulation results are consistent with Sa et al. (2014) who attempted to quantify the effect of misregistration on SV measures by performing image registration on 5 normal subjects. SV from registered images showed a slight increase in width (by 0.04) and amplitude (by 0.02) of the SV distribution on a log scale compared to nonregistered images (Sa et al. 2014). The impact of image misalignment in these simulations is greater, due to the more extreme EELV changes included. In essence we simulated a worst‐case scenario from a particularly variable SVI experiment, including all images in that scenario, while Sá et al. discarded and interpolated images that significantly deviated from FRC during imaging (>10% in plane area change).

### Assumptions and limitations

Partial pressure of deoxygenated arterial blood is an input to the gas exchange model and was held constant at 40 mmHg in these simulations. Thus, its O_2_ contribution to the MR signal resembles that of a linear offset in magnitude of the signal. Previous studies have shown that this partial pressure is altered slightly under pure oxygen breathing, to approximately 50 mmHg which is a small change compared to that in the pulmonary venous blood. Furthermore, the inclusion of deoxygenated pulmonary arterial blood (at 40 mmHg) was shown to make an insignificant contribution to the SV (see section *Contribution of pulmonary venous blood)*.

We have implemented a simplified form of image misregistration by stretching the lung ROI in the cranio‐caudal direction only. To overcome this simplification we elected to include simulated images in which the lung ROI area change exceeded the 10% threshold that would normally serve to result in deletion of that image and subsequent interpolation. Thus, we believe that although the simulation itself is simplified, results drawn from this are suggestive of the robustness of SVI to potential sources of error from using nonregistered images.

The conclusions drawn from this study are based on analysis of lung models created from two healthy young subjects with normal lung function, and may differ for subjects with impaired lung function. Healthy subjects are known to display a strong gravitational gradient in ventilation (Musch and Venegas 2005), with well‐documented fractal characteristics where regions with similar ventilation are spatially clustered (Altemeier et al. 2000), while subjects with lung disease such as asthma (Fain et al. 2008), COPD (Rodriguez‐Roisin et al. 2009) or elderly subjects (Verbanck et al. 2012) exhibit more heterogeneity that is likely not adequately captured by a single slice implementation. Furthermore, the contribution of pulmonary venous signal to measured SV distribution may be different in diseased lungs leading to higher or lower contributions to the SV signal.

## Conclusions

An integrated in silico modeling framework including 3D anatomically based tree structure, physiological models of tissue deformation, ventilation, perfusion and gas exchange was used to simulate a virtual SVI experiment. Three assumptions of the SVI technique were interrogated to assess the ability of this technique to measure SV distribution in the healthy young adult lung. Model results demonstrate that imaging a single slice is representative of overall SV gradient and distribution in the case of healthy lungs. Contribution of pulmonary venous signal to SVI measurement is relatively minor due to the fact that the SVI measurement is based on the temporal change in signal rather than absolute magnitude of signal, and is largely restricted to the appearance of voxels with an apparently high SV resulting from the large pulmonary veins. Misalignment of tissue during imaging resulted in an underestimation SV in general, although this is robust against a small extent of image misalignment.

## Ventilation

Ventilation distribution was solved following Swan et al. (2012), by coupling rigid airway with compliant acini that were both embedded within the previously described lung tissue continuum. Poiseuille flow with correction for energy losses at airway bifurcations were solved for the flow model, as:

(A4) $$P_{{\mathrm{aw}}_{2}}-P_{{\mathrm{aw}}_{1}}=Z_{\mathrm{pe}}R_{p}\dot{V}$$

where $P_{{\mathrm{aw}}_{1}}$, $P_{{\mathrm{aw}}_{2}}$ denote the pressure differences between the two nodes of an airway branch, and determines the air flow rate through the branch. $R_{p}$ is the Poiseuille resistance and is multiplied to the correction term for energy dissipation $Z_{\mathrm{pe}}$ (found in Equation (A5) in). Flow was conserved at the bifurcations.

An equation of motion relates flow at the terminal airway with the elastic acini units using:

(A5) $$P_{\mathrm{aw}}=\frac{V_{a}}{C}+R_{\mathrm{aw}}\dot{V}-P_{e}$$

Here, $P_{\mathrm{aw}}$, $R_{\mathrm{aw}}$ and $\dot{V}$ are pressure, resistance and flow of the terminal airway, respectively, and $P_{e}$ is the output from the tissue deformation model that calculates the local pressure expanding the acinus.

## Perfusion

Blood flow through the pulmonary circulation was solved using the model of Clark et al. (2011). The Poiseuille equation was used to model blood flow through the extra‐acinar pulmonary arteries and veins. For a pulmonary vessel branch with length *L* and diameter *D*, its pressure drop along the branch (ΔP) is described as follows:

(A6) $$\Delta P=\frac{128\mu L\dot{Q}}{\pi D^{4}}+\rho_{b}g\cos\theta$$

where µ is blood viscosity, $\dot{Q}$ is the blood flow rate in the branch, $\rho_{b}$ is blood density, *g* is gravitational acceleration, and θ is the angle that the vessel. Diameter of the extra‐capillary vessels is regulated by transmural pressure $P_{\mathrm{tm}}$:

(A7) $$D=\left(D_{0}\right)\left(\alpha P_{\mathrm{tm}}+1\right)$$

where *D* are the unstrained vessel diameter, and α is the vessel compliance constant valued at 1.49 × 10^−4^ Pa·sec^−1^. *P_tm_* is calculated as the sum of *P_b_* (blood pressure of the segment) and the local elastic recoil pressure, *P_e_* (local elastic recoil pressure, derived from mechanics model).

## [O_2_] Distribution Within the Airway and Vascular Networks

For this study, a previously published model (Swan and Tawhai 2011) was extended to predict the O_2_ transport and exchange within the entire lung. Similar governing equations and solution methodologies were applied except (1) gas exchange was modeled within lumped acinar units rather than distributed gas exchanging surfaces in the acinar airways in (Swan and Tawhai 2011), (2) a tissue compartment was added into the gas exchange model to capture O_2_ dissolved in lung tissue, and (3) a Lagrange‐Galerkin scheme with Peclet number based functional split was used to solve the governing transport equation for advection and diffusion in the conducting airway. This is described here in brief.

Both ventilation and perfusion in each acinar unit was assumed to be time‐invariant, and thus receive the same amount of blood and air flow throughout the breathing cycle determined by advection of air (output of time averaged ventilation model), and capillary blood volume and red blood cell (RBC) transit time for the perfusion model. A mix of advection and diffusion processes transport oxygen through the conducting airways to each acinar unit, described for the 1D advection diffusion equation in Equation (A1).

(A8) $$\frac{dc}{dt}+u\frac{dc}{dx}=D\frac{d^{2}c}{dt^{2}}$$

where *c* is the concentration of O_2_ [O_2_], *D* is the binary gas diffusion coefficient for O_2_ and N_2_ at 22.5 mm^2^·sec^−1^, *x* is geometric coordinate and *u* is the time averaged air velocity generated from the ventilation model.

A Lagrange‐Galerkin scheme with a Peclet number based functional split was used to evaluate nodal [O_2_] throughout the tree structure at each time step. The operator splitting approach solves the equation (Equation (A1)) in two steps: the advection portion using a Finite Difference approximation, and then the Galerkin finite element model (FEM) was used to solve the pure diffusion portion using the solution from step 1 as the initial condition. The functional split is applied such that solution of nodes with Peclet numbers ($P_{e}=\frac{\text{Lu}}{D})$ ≧5 were assumed to have negligible diffusion, and thus only the advective portion was solved.

A step function for flow was applied at the trachea, such that flow is a constant 0.16 L·sec^−1^ during inspiration and 0.16 L·sec^−1^ during expiration, to give a tidal volume of 650 mL and breath duration of 5 sec with an inspiratory:expiratory (I:E) ratio of 1:1. During inspiration, a zero flux boundary condition was set at the terminal bronchioles; and during expiration, the terminal bronchiole concentration equals that of acinar concentration.

Acinar volume change during the breathing cycle depends on existing acinar volume and is proportional to the amount of acinar ventilation over a time step (*δt*). Acinar concentration at each time step depends on acinar concentration and volume at the previous time step, fresh inspired gas from the conducting airway and the gas exchange flux with the capillary blood and tissue compartment over each time step during inspiration; and depends solely on gas exchange flux during expiration.

At each time step, the tissue PO_2_ (*P_t_O_2_*) and blood PO_2_ (*P_b_O_2_*) for each acinar unit was updated using:

(A9) $$\frac{dP_{t}O_{2}}{dt}=\frac{T_{\mathrm{ta}}}{v_{t}\sigma_{O_{2}}}\times \left(P_{A}O_{2}-P_{t}O_{2}\right)+\frac{T_{b}}{v_{t}\sigma_{O_{2}}}\times \left(P_{t}O_{2}-P_{b}O_{2}\right)$$

(A10) $$\frac{dP_{b}O_{2}}{dt}=\frac{T_{b}}{v_{b}\sigma_{O_{2}}}{\left(1+\frac{4\left[\text{Hb}\right]}{\sigma_{O_{2}}}\frac{dS_{O_{2}}}{dP_{b}O_{2}}\right)}^{-1}\times \left(P_{t}O_{2}-P_{b}O_{2}\right)$$

where *T_(i=ta,b)_* is the transfer factor for alveolar‐tissue and tissue‐capillary, respectively, *v_(i=ta,b)_* is the tissue and blood volume of the acinus, respectively; $\sigma_{O_{2}}$ is O_2_ solubility in tissue and blood at 1.4 × 10^−3^ mmol/L per mmHg; [H*b*] is the hemoglobin concentration, and $S_{O_{2}}$ is the oxyhemoglobin saturation.

## Appendix A:

Essential details of each component in the modeling framework are provided below. Unless otherwise indicated, the model components have been published previously, and references are given for more comprehensive coverage of the models. Table A1 displays all study‐specific model parameters used in this study. Any parameters not shown here are the same as those used in previously published studies.

**Table A1:** Study‐specific modeling parameters used in this study

Parameter name	Subject 1 (S1, female)		Subject 2 (S2, male)	
	Value	Units	Value	Units
Strahler diameter ratio for airway network	1.10	n/a	1.15	n/a
Strahler diameter ratio for arterial network	1.50	n/a	1.51	n/a
Strahler diameter ratio for venous network	1.55	n/a	1.55	n/a
Conducting airway volume	115	mL	150	mL
Flow at pulmonary circulation inlet	80,000	mm^3^	82,500	mm^3^
Pressure at main pulmonary veins	666	Pa	666	Pa
Inspiration to expiration ratio	1	n/a	1	n/a
Reference volume ratio	0.65	n/a	0.5	n/a

### Lung Tissue Mechanics

The approach of Tawhai et al. (2009) was used to solve for deformation of lung tissue under normal gravitational loading. The lung tissue was treated as a compressible, homogeneous, and isotropic material. A strain energy density function (W) was used to relate stress and strain:

(A1) $$W=\frac{\varepsilon }{2}\exp\left(aJ_{1}^{2}+bJ_{2}\right)$$

*J*_1_ and *J*_2_ are the first and second invariants of the Green‐Lagrangian finite strain tensor and *a, b*,* ε* are constants (*a* and *b* dimensionless, *ε* with units of pressure). The lung model was first uniformly expanded from a zero stress state of 50% FRC volume, then gravitational loading was incrementally added until full loading in the supine position is reached. Stress and strain at each vessel and acinus location were estimated, and relate to tissue compliance (*C*), elastic recoil pressure (*P*_e_) and acini volume through:

(A2) $$P_{e}=\left(\varepsilon e^{\gamma }\right)\left(3a+b\right)\left(\lambda^{2}-1\right)/\left(2\lambda \right)$$

(A3) $$C=\frac{\varepsilon e^{\gamma }}{6V_{0}}\left(\frac{{\left(\lambda^{2}-1\right)}^{2}3{\left(3a+b\right)}^{2}}{\lambda^{2}}+\frac{\left(3a+b\right)\left(\lambda^{2}-1\right)}{\lambda^{4}}\right)$$

The compliance equation is calculated at every time step during the breath, and parameters *a, b,* and *ε* are defined as in Equation (A1), λ is isotropic stretch, *V*_0_ is the undeformed volume. The airways, blood vessels and acini grown from step 2 in Figure 1 are “tethered” to the tissue and moved as the tissue is displaced due to gravitational loading.
